# Job retention vocational rehabilitation for employed people with inflammatory arthritis (WORK-IA): a feasibility randomized controlled trial

**DOI:** 10.1186/s12891-017-1671-5

**Published:** 2017-07-21

**Authors:** Alison Hammond, Rachel O’Brien, Sarah Woodbridge, Lucy Bradshaw, Yeliz Prior, Kate Radford, June Culley, Diane Whitham, Ruth Pulikottil-Jacob

**Affiliations:** 1 Centre for Health Sciences Research (OT), L701 Allerton, University of Salford, Frederick Road, Salford, M6 6PU UK; 2 Centre for Health and Social Care Research, Sheffield Hallam University, Montgomery House, 32 Collegiate Crescent, Sheffield, S10 2BP UK; 3 Occupational Therapy, Royal Derby Hospital, Derby, DE22 3NE UK; 4 Nottingham Clinical Trials Unit, Queens Medical Centre, University of Nottingham, NG7 2UHL, Nottingham, UK; 5 Ageing and Disability Research Unit, Queen’s Medical Centre, University of Nottingham, Nottingham, NG7 2UH UK; 6 Derby National Rheumatoid Arthritis Society branch, Derby, UK; 7 Warwick Clinical Trials Unit, University of Warwick, Coventry, CV4 7AL UK

**Keywords:** Feasibility trial, Randomized controlled trial, Arthritis, Inflammatory arthritis, Rheumatoid arthritis, Vocational rehabilitation, Occupational therapy, Employment, Work and presenteeism

## Abstract

**Background:**

Inflammatory arthritis leads to work disability, absenteeism and presenteeism (i.e. at-work productivity loss) at high cost to individuals, employers and society. A trial of job retention vocational rehabilitation (VR) in the United States identified this helped people keep working. The effectiveness of this VR in countries with different socioeconomic policies and conditions, and its impact on absenteeism, presenteeism and health, are unknown. This feasibility study tested the acceptability of this VR, modified for the United Kingdom, compared to written advice about managing work problems. To help plan a randomized controlled trial, we tested screening, recruitment, intervention delivery, response rates, applicability of the control intervention and identified the relevant primary outcome.

**Methods:**

A feasibility randomized controlled trial with rheumatoid, psoriatic or inflammatory arthritis patients randomized to receive either job retention VR or written information only (the WORK-IA trial). Following three days VR training, rheumatology occupational therapists provided individualised VR on a one to one basis. VR included work assessment, activity diaries and action planning, and (as applicable) arthritis self-management in the workplace, ergonomics, fatigue and stress management, orthoses, employment rights and support services, assistive technology, work modifications, psychological and disclosure support, workplace visits and employer liaison.

**Results:**

Fifty five (10%) people were recruited from 539 screened. Follow-up response rates were acceptable at 80%. VR was delivered with fidelity. VR was more acceptable than written advice only (7.8 versus 6.7). VR took on average 4 h at a cost of £135 per person. Outcome assessment indicated VR was better than written advice in reducing presenteeism (Work Limitations Questionnaire (WLQ) change score mean: VR = −12.4 (SD 13.2); control = −2.5 (SD 15.9), absenteeism, perceived risk of job loss and improving pain and health status, indicating proof of concept. The preferred primary outcome measure was the WLQ, a presenteeism measure.

**Conclusions:**

This brief job retention VR is a credible and acceptable intervention for people with inflammatory arthritis with concerns about continuing to work due to arthritis.

**Trial registration:**

ISRCTN 76777720. Registered 21.9.12.

**Electronic supplementary material:**

The online version of this article (doi:10.1186/s12891-017-1671-5) contains supplementary material, which is available to authorized users.

## Background

Work problems are common in people with inflammatory arthritis. A third of employed people with rheumatoid arthritis (RA) stop working within three years of diagnosis due to arthritis (i.e. become work disabled) and 50% within 10 years [1]. Before this, people experience work instability, i.e. a mismatch between abilities and job demands which threatens employment [2]. This is associated with increased: absenteeism, as employed people with RA average 40 days sick leave per year compared to the 6.5 days United Kingdom (UK) average [1]; and presenteeism (i.e. reduced at-work productivity), with 24% of working time lost due to health problems [3]. Once unemployed, people with RA are unlikely to return to work [4]. Many factors influence work instability and disability including: greater pain, hand pain and fatigue; unadapted work environments and equipment; physically demanding jobs; poor work self-efficacy; job strain; and limited use of self-management strategies [4–8]. Job retention vocational rehabilitation (VR) has the potential to prevent or postpone work disability through modifying such factors. For example, those with workplace ergonomic modifications are 2.5 times less likely to stop work [6]. VR should be provided early, when work instability develops. However, many with arthritis lack access to VR.

A recent systematic review identified only two successful randomized controlled trials (RCTs) of job retention VR in arthritis [9]. These demonstrated: at 4.5 years, significant reductions in job loss (*n* = 242) [10]; and at six months, improvements in work instability, work satisfaction, pain and self-perceived ability to manage work (*n* = 32) [11]. Allaire et al. [10] evaluated a brief (three hour) intervention delivered by rehabilitation counsellors in the United States of America (USA), including work assessment and problem-solving participants’ priority difficulties. It is unclear if similar results would occur in the UK, with a different social security system, employment law and rehabilitation services. Macedo et al. [11], in a UK study, evaluated a comprehensive functional, work and psychosocial assessment, followed by six to eight sessions (30–120 min each) of individualised VR and comprehensive occupational therapy, including a group patient education programme. The drive for efficiency in the UK National Health Service (NHS) means many therapy services now provide brief interventions and group education provision is patchy. Thus a longer intervention may not be feasible. Neither study measured effects on absenteeism or presenteeism. These findings suggest brief job retention VR, based on that developed in the USA [10], delivered by occupational therapists with job retention VR training as part of Rheumatology NHS services, could be effective in the UK.

The aims of this study were to determine the feasibility of conducting a full RCT of job retention VR for employed people with inflammatory arthritis, specifically RA, undifferentiated inflammatory arthritis (IA) and psoriatic arthritis (PsA), as these are common conditions seen in Rheumatology departments. We investigated: screening and recruitment processes, study uptake, barriers to participation and retention rates; treatment fidelity; feasibility of assessment methods (proportion of missing data); the most feasible primary work outcome measure (time to job loss; employment status; absenteeism; or presenteeism); estimate a sample size for a definitive RCT; proof of principle by describing any changes in work and health outcomes; and the feasibility of Rheumatology occupational therapists delivering the VR.

## Methods

### Design and setting

We conducted a feasibility RCT comparing job retention VR (plus work self-help information) with work self-help information only in six Rheumatology occupational therapy services in England from November 2011 to July 2013. The trial was managed by Nottingham Clinical Trials Unit (CTU). Ethics approval was granted by the National Research Ethics Committee East Midlands (Nottingham 1:11/EM/0103) and University of Salford Ethics Panel.

This was a three-year study. The recruitment period was seven to ten months (i.e. varying between sites). This was reduced from 12 months because of delays in study approvals and restricted availability of research facilitators (i.e. research nurses and others employed by National Institute of Health Research (NIHR) Clinical Research Networks (CRN) and NHS Trust Research and Development departments to support study recruitment in the NHS. They are not part of the research project team and thus not supervised by the trial manager). As a result, the funder (Arthritis Research UK) recommended that the follow-up period was reduced from 12 to nine months, in order to maximise the recruitment time available. The protocol was accordingly amended to conduct follow-up at nine months.

### Eligibility criteria

Participants were eligible if they were: 18 years of age or over; diagnosed with RA, PsA or undifferentiated IA (i.e. persistent symmetrical synovitis without any other known cause, but the person does not yet meet diagnostic criteria for RA); in paid work (full- or part-time); able to read, write and understand English; and willing to receive VR. We also tested which criterion best established need for VR: scoring ≥10 on the RA-Work Instability Scale (RA-WIS) [11], (i.e. a score indicating medium to high risk of work instability and need for VR [2]) or stating “Yes” to “Do you have any concerns about your health affecting your ability to work over the next few years?” [10].

Exclusion criteria were: on extended sick leave (i.e. >three months) or unemployed (including not normally in paid employment or student); within the next 12 months, either planning to retire or take early retirement (through choice or ill health), move out of area or expecting joint replacement surgery; or already receiving or awaiting VR services.

### Recruitment procedures

NIHR CRN support for screening and recruitment was agreed prior to trial start. Screening was planned to be conducted by research facilitators in at least two Rheumatology out-patient clinic sessions per week at each site. If possible, a week before clinics, the healthcare team identified working age patients with RA, PsA or IA on clinic lists and research facilitators mailed out study information packs. If not, the health care team identified patients during clinic appointments and introduced them to the research facilitator. Research facilitators then screened patients in clinic. For those eligible and willing, research facilitators explained the study, obtained written consent, recorded baseline demographic data and the participant either completed the baseline questionnaire in clinic or later at home and mailed it back to the research facilitator. Research facilitators also explained how to complete a monthly calendar reporting work status and sickness absence (see Outcome Measures: absenteeism). For those receiving study information in advance, research facilitators consented patients that day in clinic. For those referred in clinic, eligible patients were allowed at least 48 h to consider participation. These patients were then telephoned by research facilitators to complete consent via telephone and mail and to remind about baseline questionnaire return.

### Randomisation and allocation concealment

Following baseline questionnaire return, research facilitators randomized participants using a web-based randomisation system to receive either work self-help information only (control group) or VR plus work self-help information (intervention group). The randomization sequence was created using Stata 9.09.2 (Ralloc function by Philip Ryan v3.3.2) statistical software and was stratified by site, to ensure even occupational therapists’ workloads, with a 1:1 allocation using permuted blocks of randomly varying sizes. Treatment assignment was by the web-based randomisation system managed by Nottingham CTU. Following randomisation, research facilitators mailed the participant’s screening checklist, demographic record and baseline questionnaire to the trial manager, who then mailed the participant the work self-help information. Intervention group participants’ contact details were sent to the treating occupational therapist by an automatically generated e-mail from the randomization system. The research facilitator, trial manager, and research staff collecting, entering and analysing data were blinded to group allocation. Blinding of participants and therapists to trial arm was not possible. Occupational therapists were asked to continue with their usual VR practice with non-trial participants (see below).

### Control group

NHS “usual care” for work problems for most people with RA, PsA or IA is limited. Referral to occupational therapy for VR is often patchy or non-existent. VR (when provided) consists of, on average 45 [IQR 30–90] minutes work advice (without a structured work assessment), provision of work advice booklets and signposting to other services [12, 13]. Control participants therefore received written self-help work information only, i.e. a similar control to that used by Allaire et al. [10]. This consisted of a cover letter, self-help flowchart and two work advice booklets [14, 15]: see Additional file 1). Participants continued to receive usual Rheumatology care (i.e. regular out-patient appointments, prescribed medication, and referral by Consultants to therapy services as necessary).

### Intervention group

Intervention group participants also received written work self-help information and usual care. Treating occupational therapists received three days VR training [12]. Participants were seen within four weeks of referral. Direct VR consisted of up to 4.5 h of 1:1 meetings, starting with a structured work interview and ending with a telephone review. Up to 1.5 h extra contact was also possible. We planned VR to be longer than in Allaire et al.’s trial [10] as: therapists were providing this VR for the first time; Allaire et al. had recommended extra time for complex cases and a telephone review [10]; and we included optional work site visits. We paid for up to 10 h occupational therapy time (plus mileage costs for any visits) per VR group participant. This included both direct and indirect VR (i.e. non-contact time when therapists: completed treatment notes; identified solutions for work problems; liaised with team members, other agencies and employers; and travel time).

Meetings were at mutually agreed times (often early or late in the day to fit around participants’ work commitments) and locations (the occupational therapy department, participant’s home or workplace) spread over two to four months. The intervention started with a structured work interview and job discussion (i.e. an assessment of the person’s job, roles and responsibilities in relation to their condition, disease severity and activity limitations) and detailed assessment of work barriers. This was conducted using the UK Work Experience Survey-Rheumatic Conditions (WES-RC) [16–19]. This was followed by mutually agreeing priority work problems, action planning, and then a tailored, individualised programme including self-management at work, job accommodations, employment rights information and other strategies as relevant. Participants were offered a work site visit, if this was identified as relevant to their needs. VR also emphasised participants’ responsibilities in liaising with employers and included role play, as necessary, to enhance confidence requesting job accommodations (See Additional file 1).

### Treatment fidelity

The WORK-IA VR Resource Manual was provided to support VR delivery. (See Additional file 1) [20]. Monitoring visits assessed treatment fidelity conducting the WES-RC, problem identification and treatment planning. Additionally, a random six WES-RCs and treatment notes were reviewed for problem identification and matching treatment to problems.

### Outcome measures

Follow-up data was collected using a questionnaire booklet at six and nine months, mailed by the trial manager. After two weeks, if not returned, a telephone reminder was given and a second questionnaire mailed, if necessary. The following outcomes and information were collected at each time point (baseline, six and nine months):

Demographic and work data: age, gender, condition duration, marital and living status (at baseline only); medication regimen; current job(s); years in current main job; whether disclosed arthritis to employer and/or work colleagues; and number of normal working hours.

#### Work measures

Employment status: whether in full- or part-time work, on long term sick leave, or stopped working (with date when stopped and reason).

Presenteeism: these measures evaluate the effect of a condition on the quality or quantity of work productivity [21]. Three measures evaluated different aspects:

i) RA- Work Instability Scale (RA-WIS): 23 true/false items measuring, at present, the degree of mismatch between functional abilities and workplace demands [2].
ii) Work Activities Limitations Scale (WALS): 12 items (time frame unspecified) indicating degree of difficulty performing physical work activities, time, mental and output demands (0 = no difficulty; 3 = unable to do), with additional items for whether help or equipment are used for each [22].
iii) The Work Limitations Questionnaire (WLQ): 25 items, indicating the amount of time, in the last two weeks, a person was limited in: physical work demands, time demands, mental-interpersonal demands and output demands. The four sub-scale scores and Summed score (i.e. average of the four sub-scales) range from 0 to 100%. A percentage Productivity Loss score can also be calculated [23].

Work self-efficacy: confidence in ability to continue working with arthritis; and ability to manage arthritis at work (0–10 numeric rating scales (NRS)).

Satisfaction with work rehabilitation advice received (0–10 NRS at six months only).

#### Health related outcomes

SF-12v2 Health Survey: 12 items, assessed over the last four weeks, scored as physical and mental health sub-scales [24].

Multi-dimensional Health Assessment Questionnaire (HAQ): the modified HAQ (eight activity limitation items); psychological status HAQ (four items); pain, fatigue and a global rating of health measured using 100 mm Visual Analogue Scales (VAS), assessed over the last week [25].

Hand/ wrist pain: pain in the last week during moderate activity (100 mm VAS).

Euroqol five dimensions questionnaire (EQ5D): measuring quality of life [26].

#### Health economic outcomes

A self-report measure was resource use questionnaire (use of health resources, health-related transport costs, personal and domestic care support, work support and adaptations).

#### Work status measures

In addition to the questionnaire booklet, participants filled out a monthly tear-off calendar, to record their work status each day (i.e. performed paid work, unable to perform paid work due to arthritis, unable to perform paid work due to other reasons, day off). This was modified from Part 1 of the Health and Labour Questionnaire [27]. Participants were asked to return each page at month end in Freepost envelopes to the trial manager. If not returned within two weeks, the trial manager telephoned to remind return. The following work outcomes were derived from the monthly calendar:

Time to temporary or permanent job loss (days): recording on which date their contract ended, if they stopped working and, if a new job was obtained, their contract started.

Absenteeism: over nine months, the number of days sickness absence attributable to either arthritis or other causes (e.g. common ailments), not including days not normally worked.

Participants were not telephoned to obtain missing data from returned questionnaires or minimal datasets from non-responders, apart from for the absenteeism measure.

#### VR provision

Therapists completed a VR Treatment Record of: duration of treatment contact for direct VR (i.e. with participant) and indirect VR (e.g. administration, making referrals, sourcing information, treatment planning, travel time for home/ work visits); treatment location; and travel mileage.

### Sample size

A statistically based sample size estimate was inapplicable for this feasibility study. Randomising 100 participants would permit estimation of the percentage of overall drop-outs to +/− 10% points at most, with 95% confidence. Drop-outs were considered those who did not attend VR or did not return follow-up questionnaires.

### Statistical analyses

Analyses were mainly descriptive in order to determine if a definitive RCT is feasible. Recruitment and retention rates were summarised descriptively. For each outcome measure and trial arm, the proportion of missing data was described and the outcome at follow-up summarised (using means and standard deviations for continuous measures or frequency counts and percentages for categorical data). Where, applicable, change from baseline was also summarised and effect sizes were calculated) as mean change/standard deviation at baseline) to compare the internal responsiveness of outcomes. An effect size of 0.2 is interpreted as small, 0.5 medium and 0.8 large effect sizes [28]. All analyses were according to randomisation allocation. Quality of Adjusted Life Years (QALYs) were calculated using EQ5D. VR duration and costs were identified from the VR Treatment Records and costed using published data [29].

## Results

### Project schedule, screening, recruitment and retention rates

Ethics approvals were obtained to schedule. Sites recruited varyingly between November 2011 and September 2012 and actively recruited for 6.5 months (1QR 5–7) (i.e. 54% of the original 12 months planned time). This was because Local NHS Trust Research and Development department (R&D) approvals were delayed and took until month 14 at five sites and month 17 at one site. Five sites were re-scheduled to open for ten and one for seven months. Average time to recruit the first patient was 14 weeks (IQR 10.5–17.25) as, for the first three to four months, there was little or no research facilitator availability at four sites. At six months, two sites closed: research facilitator support was withdrawn at one and the only occupational therapist went on long-term sick leave at the other.

**Fig. 1:**
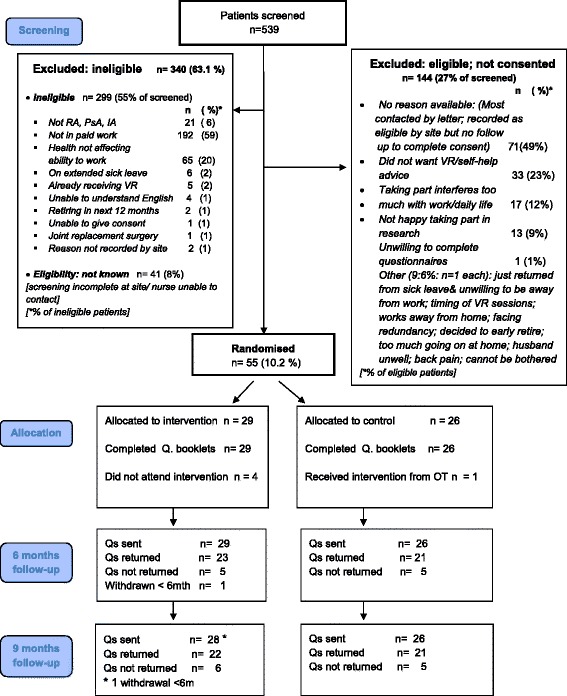
CONSORT flow diagram

The flow of participants is shown in Fig. 1. Of the 539 screened, 37% (*n* = 199) were eligible. Of these, 28% of those eligible (*n* = 55; 10% overall) consented. At screening, most had RA-WIS scores ≥10 (i.e. moderate to high risk of work disability). Only six (11%) had scores <10 but answered “yes” to concerns about continuing working with arthritis. Their average RA-WIS score at screening was 5.5 (SD 2.7) but rose to 7.8 (SD3.7) at baseline, as three now reported RA-WIS scores ≥10. The two intervention group participants with scores <10 at screening, had multiple problems identified in the WES-RC, indicating VR was applicable. Given most people with concerns had RA-WIS scores ≥10, and those scoring <10 at screening had scores close to or ≥10 at baseline, using the concerns criterion is applicable for a future trial. Most participants completed baseline questionnaires at home, with only 10 choosing to do so in clinic. Within the two groups, 1/26 in the control group was incorrectly referred to occupational therapy by the CTU and received VR; and 4/29 in the intervention group did not complete VR (one was not referred by the CTU; one had already retired early; one did not want VR; and one could not be contacted). By six months, one person in the intervention group withdrew.

However, 72% (*n* = 144; or 27% overall) of those eligible did not consent. Almost half (*n* = 71; 13% overall) were recorded as “no reason given” for non-participation. The trial manager identified these were often people needing time to consider participation after screening but some research facilitators were unable to contact patients by telephone to complete consent. Research facilitators worked during the day (when most participants were at work) and not evenings, when patients could be more readily contactable. The other half of patients (*n* = 73) not consenting gave understandable reasons (see Fig. 1). Of these 12% (*n* = 17) thought VR would interfere too much with their work/ daily life and 23% (*n* = 33) did not want VR/work advice. During the trial, the trial manager identified some research facilitators were unclear about flexibility of the VR and employer contact being unnecessary. To help research facilitators allay patients concerns, further site training was provided.

Overall, 63% (*n* = 340) were ineligible. Of these, 8% (*n* = 41) of patients had incomplete screening. The trial manager identified these were patients sent invitation letters prior to clinics, added to the screening list but some research facilitators were then unable to attend clinic to complete screening in person or contact patients later by telephone.

### Participants’ baseline characteristics

The two groups were well balanced at baseline across most demographic, work and health measures (Tables 1 and 2). However, there were more people with level 3 and 4 jobs in the intervention group compared to level 2 jobs in the control group (Table 1). The baseline RA-WIS score was 15.7 (SD 5.3), with six having low (<10), 20 moderate (≥10 to <17) and 29 high (≥17) work instability.

**Table 1:** Baseline demographic characteristics (*n* = 55)

Characteristic	Intervention (*n* = 29)	Control (*n* = 26)	Total (*n* = 55)
Age (years):Mean [sd]	47.7 [10.4]	50.5 [6.4]	49.0 [8.8]
Gender: Females n (%)	22 (75.9%)	20 (76.9%)	42 (76.4%)
Marital status:			
Married/living with partner	24 (82.8%)	16 (61.5%)	40 (72.7%)
Other	5 (17.2%)	10 (38.5%)	15 (27.3%)
Living alone: n (%)	5 (17%)	9 (35%)	14 (25%)
Type of arthritis: n (%)			
Inflammatory arthritis	5 (17.2%)	3 (11.5%)	8 (14.5%)
Rheumatoid arthritis	17 (58.6%)	17 (65.4%)	34 (61.8%)
Psoriatic arthritis	7 (24.1%)	6 (23.1%)	13 (23.6%)
Disease duration (years): Median [IQR]	5.0 [0.7–10.0]	5.3 [1.0–17.0]	5.0 [0.7–12.0]
Currently taking oral or intramuscular steroids: Yes n (%)	12 (41.4%)	7 (26.9%)	19 (34.5%)
Currently taking disease modifying drugs: Yes n (%)	26 (89.7%)	20 (76.9%)	46 (83.6%)
Currently taking biologic drugs: Yes n (%)	8 (27.6%)	9 (34.6%)	17 (30.9%)
Main job type (SOC2010 major group)*			
Level 4 (Professional and managerial)	12 (41%)	3 (12%)	15 (27%)
Level 3 (Associated professional and technical/ skilled trades)	7 (24%)	1 (4%)	8 (16%)
Level 2 (Administrative, caring, leisure, sales, customer service; process, plant and machinery operatives)	8 (28%)	21 (81%)	29 (53%)
Level 1 (Elementary occupations)	2 (7%)	1 (4%)	3 (5.5%)
Years in current main job: Mean [sd]	11.0 [8.9]	13.5 [9.7]	12.2 [9.3]
Full-time work (>35 h/week) n (%)	17 (58.6%)	16 (61.5%)	33 (60%)
Average hours worked/week: Mean[sd]	32 [9.1]	32.8 [8.7]	32.4 [8.8]
Disclosed arthritis to: Yes n (%)			
Employer	25 [86.2%]	23 [88.5%]	48 [87.3%]
[At least some] Work colleagues	26 [90%]	23 [88.5%]	49 [90%]

Key: * SOC2010 = Standard Occupational Classification 2010 [56]

**Table 2:** Work and health related characteristics at baseline (*n* = 55)

Characteristic	Intervention (*n* = 29)	Control (*n* = 26)	Total (*n* = 55)
RA-Work Instability Scale (0–23): Mean [sd]	16.2 [5.0]	15.0 [5.7]	15.7 [5.3]
Workplace Activity Limitations Scale (0–36): Mean [sd]	12.8 [5.3]	12.7 [6.2]	12.7 [5.7]
Workplace Limitations Questionnaire (0–100):			
*Productivity Loss: Mean [sd]*	10.9 [4.9] (*n* = 27)	11.2 [5.6] (*n* = 24)	11.0 [5.2] (*n* = 51)
*Time management demands: Mean [sd]*	52.1 [25.1] (*n* = 28)	51.9 [28.9] (*n* = 25)	52.0 [26.7] (*n* = 53)
*Physical demands: Mean [sd]*	45.1 [23.4]	42.8 [23.1]	44.0 [23.1]
*Mental/interpersonal demands: Mean [sd]*	32.5 [21.8]	31.7 [24.4]	32.1 [22.8]
*Output demands: Mean [sd]*	40.0 [27.5] (*n* = 28)	41.0 [28.0] (*n* = 25)	40.5 [27.5] (*n* = 53)
*WLQ Summed: Mean [sd]*	43.0 [18.2] (*n* = 27)	43.9 [20.1] (*n* = 24)	43.4 [18.9] (*n* = 51)
Work Self-Efficacy: Mean[sd] (0–10)	7.0 [2.4]	7.2 [2.2]	7.1 [2.1]
Confidence managing arthritis at work: Mean[sd] (0–10)	4.6 [2.7]	5.7 [2.4]	5.1 [2.6]
SF12v2 Health Survey (0–100):			
*Physical component:* Mean [sd]	32.5 [8.3]	32.6 [10.1]	32.5 [9.1]
*Mental component:* Mean [sd]	40.1 [10.9]	42.4 [12.4]	41.2 [11.6]
Multidimensional Health Assessment Questionnaire (0–3):			
*Functional status: Mean [sd]*	0.6 [0.4]	0.7 [0.5]	0.7 [0.5]
*Psychological status: Mean [sd]*	1.0 [0.6]	0.9 [0.7]	1.0 [0.7]
Pain VAS (0–100): Mean [sd]	50.4 [22.6]	45.7 [25.7]	48.2 [24]
Hand Pain VAS (0–100): Mean [sd]	51.8 [26.4]	51.3 [27.3]	51.6 [26.6]
Fatigue VAS (0–100): Mean [sd]	60.0 [22.4]	58.7 [30.1]	59.4 [26.1]
Perceived Health Status VAS (0–100): Mean [sd]	50.9 [18.4]	48.7 [23.1]	49.9 [20.6]
EQ5D VAS Health State mean [sd]	55.7 [16.9]	61.0 [18.7]	58.2 [17.8]

Key: For all variables higher scores indicate worse work/health status, apart from work self-efficacy where higher scores indicate increased confidence and SF12v2 measures where higher scores indicate better health status

### Fidelity of VR delivery

The monitoring visits confirmed occupational therapists conducted assessments appropriately. The WES-RC review indicated therapists identified a wide range of problems and provided appropriate treatment matching problems. The commonest problems were: Preparing for/getting to work (Getting ready in the morning, *n* = 17) and Driving to work, *n* = 13); Physical Demands (Standing too long, *n* = 14; Lifting/moving, *n* = 14); Work/mental time demands (Concentrating, *n* = 12; Work pace scheduling, *n* = 12). Matching solutions included, respectively: advance planning, daily activities and driving advice; pacing and moving and handling advice/ training; and fatigue management.

### Feasibility of assessment methods

At baseline, most (*n* = 53) participants in both groups correctly completed all questionnaire items, apart from two (one in each group) having missing data in either the output demands or time management demands scales of the WLQ. At six and nine months, 44/55 and 43/55 respectively returned questionnaires (80% and 78% response rates: see Fig. 1). Within questionnaires, 75 to 80% of participants in both groups fully completed measures. In the monthly absenteeism calendars, 80% in both groups completed at least 70% of monthly forms. For the EQ-5D and the resource questionnaire, used for health economic analysis, it was feasible to collect the required quality of life and cost data. We identified “rheumatology appointments” needed separating into rheumatology consultant or nurse appointments, due to differing costs.

### Outcomes

At nine-month follow-up, for the intervention group, changes in score from baseline corresponded to medium effect sizes (+/− 0.5 to +/− 0.7) in the RA-WIS, WLQ Summed and Productivity Loss scores, confidence in managing arthritis at work, physical function (SF-12v2), pain, hand pain and perceived health status (Tables 3 and 4). In comparison, there were smaller changes in scores for the control group, corresponding to either no or small effect sizes (Tables 3 and 4). Two participants (both in the intervention group) were known to have stopped working (one had already taken early retirement before referral for VR). Similar numbers of people in both groups had sickness absences at six and nine-month follow-up, although the percentage of working days lost was less in the intervention group at both time points (Table 5). Satisfaction with work advice received was higher in the intervention group (7.8 (SD 2.1)) versus 6.7 (SD 2.3) in the control group. No adverse events were reported.

**Table 3:** Work and health related outcomes at six and nine months from questionnaires

Outcome	Six months		Nine months	
	Intervention (*n* = 23)	Control (*n* = 21)	Intervention (*n* = 22)	Control (*n* = 21)
RA-Work Instability Scale (0–23): Mean [sd]	13.1 [6.3] (*n* = 22)	15.5 [5.8] (*n* = 21)	12.3 [7.8] (*n* = 21)	14 [6.2] (*n* = 21)
Workplace Activity Limitations Scale (0–36): Mean [sd]	10.7 [6.5] (*n* = 22)	13.2 [6.4] (*n* = 21)	10.4 [6.8] (*n* = 21)	13.5 [9.0] (*n* = 21)
Workplace Limitations Questionnaire (0–100):				
*Productivity Loss: Mean [sd]*	9.3 [5.8] (*n* = 21)	10.3 [4.6] (*n* = 19)	7.5 [5.3] (*n* = 20)	9.3 [6.3] (*n* = 20)
*Time management demands: Mean [sd]*	39.3 [29.6] (*n* = 22)	47.1 [22.8] (*n* = 19)	33.3 [26.1] (*n* = 21)	41.8 [28.4] (*n* = 20)
*Physical demands: Mean [sd]*	36.6 [18.6] (*n* = 22)	46.3 [24.5] (*n* = 21)	35.7 [26.9] (*n* = 20)	37 [18.9] (*n* = 21)
*Mental/interpersonal demands: Mean [sd]*	31.5 [23.2] (*n* = 22)	32.5 [18] (*n* = 20)	25.8 [20.1] (*n* = 21)	28.5 [23.4] (*n* = 21)
*Output demands: Mean [sd]*	36 [28] (*n* = 21)	41.3 [24.6] (*n* = 20)	25.2 [23.3] (*n* = 21)	38.5 [32.2] (*n* = 21)
*WLQ Summed: Mean [sd]*	35.4 [21.1] (*n* = 21)	41.3 [18.1] (*n* = 19)	29.8 [19.7] (*n* = 20)	35.9 [21.5] (*n* = 20)
Work Self-Efficacy: Mean[sd] (0–10)	7.6 [2.2] (*n* = 22)	6.5 [2.8] (*n* = 21)	7.8 [2.1] (*n* = 21)	6.5 [2.2] (*n* = 21)
Confidence managing arthritis at work: Mean[sd] (0–10)	7.1 [2.5] (*n* = 22)	6.1 [2.7] (*n* = 21)	7.5 [2.1] (*n* = 21)	6.4 [2.6] (*n* = 21)
SF12v2 Health Survey (0–100):				
*Physical component:* Mean [sd]	37.7 [10.1]	32.4 [10.6]	38.7 [10.5]	34.3 [10.9]
*Mental component:* Mean [sd]	41.3 [12.8]	42 [9.5]	41.6 [12.5]	44.2 [11.4]
Multidimensional Health Assessment Questionnaire (0–3):				
*Functional status: Mean [sd]*	0.6 [0.5]	0.8 [0.5]	0.6 [0.6]	0.7 [0.6]
*Psychological status: Mean [sd]*	0.9 [0.7]	1.0 [0.5]	0.9 [0.7]	0.9 [0.6]
Pain VAS (0–100): Mean [sd]	43.1 [27.1]	48.5 [27.3]	40.5 [27.6]	44.1 [27.0]
Hand Pain VAS (0–100): Mean [sd]	43.0 [31.0]	52 [28.9]	39.8 [30.1]	52.3 [27.1]
Fatigue VAS (0–100): Mean [sd]	54.4 [30.2]	57.7 [21.9]	54.3 [33.3]	58.7 [23.8]
Perceived Health Status VAS (0–100): Mean [sd]	41 [26.5]	52 [22.2]	38.5 [25.2]	44.2 [23.7]
EQ5D VAS Health State mean [sd]	63.2 [23.4]	63.1 [20.1]	59.1 [26.0]	60.4 [22.4]

Key: For all variables, higher scores indicate worse work/health status, apart from work self-efficacy where higher scores indicate increased confidence and SF12v2 measures where higher scores indicate better health status

One participant in the intervention group who returned the questionnaire booklets stopped working during the 6-month follow-up period and did not complete the presenteeism outcomes at either timepoint. At nine months, pain, hand pain, fatigue and perceived health status VAS were not completed by one participant in the control group, who returned the questionnaire

**Table 4:** Outcome measures: changes (mean [sd]) at six and nine months from baseline, with effect sizes

	Six months – baseline				Nine months - baseline			
Outcome	Intervention (*n* = 23)	Effect size	Control (*n* = 21)	Effect Size	Intervention (*n* = 22)	Effect Size	Control (*n* = 21)	Effect size
Presenteeism:								
RA-Work Instability Scale (0–23): Mean [sd]	−2.7 [4.0] (*n* = 22)	−0.5	0 [3.4] (*n* = 21)	0	−3.9 [5.3] (*n* = 21)	−0.7	−1.0 [3.5] (*n* = 21)	−0.2
Workplace Activity Limitations Scale (0–36): Mean [sd]	−1.6 [4.2] (*n* = 22)	−0.3	0.2 [30.1] (*n* = 21)	0.04	−2.2 [4.5] (*n* = 21)	−0.4	0.6 [6.0] (*n* = 21)	0.1
Workplace Limitations Questionnaire (0–100):								
*Productivity Loss: Mean [sd]*	−0.7 [3.5] (*n* = 21)	−0.1	0.4 [2.7] (*n* = 19)	0.07	−3.2 [3.7] (*n* = 20)	−0.6	−0.4 [4.3] (*n* = 20)	0.1
*Time management demands: Mean [sd]*	−11.0 [26.4] (*n* = 22)	−0.4	−1.9 [15.1] (*n* = 19)	−0.07	−17.5 [21.9] (*n* = 21)	−0.7	−3.8 [20.3] (*n* = 20)	−0.1
*Physical demands: Mean [sd]*	−6.8 [23.1] (*n* = 22)	−0.3	2.63[20.1] (*n* = 21)	0.1	−9.0 [26.0] (*n* = 20)	−0.4	−5.9 [17.5] (*n* = 21)	−0.3
*Mental/interpersonal demands: Mean [sd]*	0.8 [16.3] (*n* = 22)	0.04	0.39[14.5] (*n* = 20)	0.02	−5.7 [18.0] (*n* = 21)	−0.3	−1.6 [15.0] (*n* = 21)	−0.1
*Output demands: Mean [sd]*	−1.0[20.2] (*n* = 21)	−0.03	1.2[13.4] (*n* = 20)	0.04	−15.2 [21.5] (*n* = 21)	−0.6	−0.07[23.0] (*n* = 21)	−0.03
*WLQ summed*	−4.7 [13.9] (*n* = 21)	−0.3	1.2 [10.8] (*n* = 19)	0.06	−12.4 [13.2] (*n* = 20)	−0.7	−2.5 [15.9] (*n* = 20)	−0.1
Confidence related to work:								
Work Self-Efficacy: Mean [sd] (0–10)	0.3 [2.1]	0.2	−0.5 [2.0]	- 0.2	0.5 [2.2]	0.3	−0.3 [1.6]	−0.2
Confidence managing arthritis at work: Mean[sd] (0–10)	0.6 [2.4]	0.3	−0.3 [1.9]	−0.1	1.1 [2.5]	0.5	−0.5 [1.9]	−0.2
Health status:								
SF12v2 Health Survey (0–100):*								
*Physical component:* Mean [sd]	4.5 [10.4]	0.5	0.7 [6.0]	0.08	5.3 [11.1]	0.6	2.5 [8.3]	0.3
*Mental component:* Mean [sd]	0.9 [7.7]	0.08	−1.6 [7.6]	−0.1	2 [7.8]	0.2	−0.5 [8.9]	−0.04
Multidimensional Health Assessment Questionnaire (0–3):								
*Functional status: Mean [sd]*	0 [0.5]	0	0 [0.4]	0	0 [0.5]	0	0 [0.4]	0
*Psychological status: Mean [sd]*	−0.1 [0.5]	−0.3	0.2 [0.4]	0.3	−0.2 [0.6]	−0.3	0 [0.4]	0
Pain VAS (0–100): Mean [sd]	−7.0 [33.7]	−0.3	5.1 [22.9]	0.2	−12.0 [27.0]	−0.5	0.1 [21.8]	0
Hand Pain VAS (0–100): Mean [sd]	−9.7 [34.5]	−0.4	−3.0 [20.1]	−0.1	−12.5 [32.6]	−0.5	−0.9 [22.1]	−0.03
Fatigue VAS (0–100): Mean [sd]	−5.2 [23.7]	−0.2	0.5 [25.2]	0.02	−5.3 [22.9]	−0.2	5.4 [26.4]	0.2
Perceived health status VAS (0–100): Mean [sd]	−9.3 [25.7]	−0.5	3.9 [22.7]	0.2	−14.6 [28.8]	−0.7	−3.9 [15.8]	−0.2
EQ5D VAS Health State mean [sd]	6.96 [23.23]	0.4	0.57 [19.54]	0.03	3.52 [28.89]	0.2	−0.43 [17.62]	−0.02

Key: For all variables negative change and ES scores indicate better outcomes, apart from work self-efficacy, SF12v2 and EQ5D health state measures where positive change and ES scores indicate better outcomes

One participant in the intervention group who returned the questionnaire booklets stopped working during the 6-month follow-up period and did not complete the presenteeism outcomes at either timepoint. At nine months, pain, hand pain, fatigue and perceived health status VAS were not completed by one participant in the control group, who returned the questionnaire

**Table 5:** Employment status and absenteeism at six and nine months (*n* = 55)

Characteristic	Six months: Intervention (*n* = 23)	Six months: Control (*n* = 21)	Nine months: Intervention (*n* = 22)	Nine months: Control (*n* = 21)
Employment status:				
*Working full time*	15	11	13	11
*Working part-time*	7	8	7	8
*On sick leave*	0	2	0	2
*Stopped working*	1	0	2	0
Sickness absence:	*n* =24	*n* =21	*n* =21	*n* =19
*Sickness absence any reason (no.):*	18 (75%)	16 (76.2%)	17 (81.1%)	16 (84.2%)
*Sickness absence due to arthritis (no.)*	12 (50%)	13 (61.9%)	14 (66.7%)	14 (70.0%)
*Percentage working days lost due to sickness (any reason): Mean [SD]*	8.4 [11.7]	18.6 [25.8]	9.6 [13.6]	20 [27.1]
Median [IQR]	3.1 [0.5-10.7]	8.2 [2.8-33.6]	4.4[ 0.7-13.1]	8.3 [2.1-29.3]
*Percentage working days lost due to arthritis:Mean [SD]*	5.5 [11.2]	12.4 [23.6]	8.0 [13.8]	15.0 [25.0]
Median [IQR]	0.5 [0-4.4]	3.5 [0-9.1]	2.5 [0-7.9]	5 [0-21.4]

Participants in the intervention group attended on average 2.86 (SD 1.55) VR sessions (*n* = 25 participants attending VR). Direct VR lasted on average 3.92 (SD 1.61) hours and indirect VR was an additional 1.68 (SD 1.46) hours. The average cost per participant of VR was £135.18 (SD £81.80) for occupational therapy staff time and mileage costs.

Mean EQ-5D health state changes in scores from baseline to six and nine months corresponded to small effect sizes in the intervention group and none in the control group (Tables 2, 3 and 4). For the EQ5D-3 L mean scores were similar between groups: intervention was 0.56 (baseline), 0.64 (6 months) and 0.56 (9 months) and control group was 0.52 (baseline), 0.56 (6 months) and 0.57 (9 months). QALYS were −0.60 for the intervention group and −0.56 for the control group, i.e. the intervention group gained 0.05 more QALY than the control group. Hospital resource use was similar at baseline, six and nine months between the two groups, as was help from others with home activities (see Additional file 1: Table S6).

### Choice of primary outcome measure

Participants interviewed about the outcome measures (*n* = 8) reported no difficulties completing presenteeism or absenteeism measures and expressed no preference for the most important to use. Completion rates were better for presenteeism than absenteeism measures. The three presenteeism measures (RA-WIS, WLQ, WALS) had similar psychometric properties, correlated similarly with other health and work measures and had similar completion rates. Larger effect sizes were seen for the WLQ Summed score and RA-WIS (i.e. +/− 0.7 at nine months, see Table 4), suggesting these measures would more likely detect differences in a trial. Very few stopped working within the nine-month follow-up period (*n* = 2), suggesting time to job loss and employment status are not applicable outcomes unless a trial has a long follow- up (e.g. four to five years).

## Discussion

This feasibility trial demonstrated job retention VR was credible, acceptable and deliverable to people with RA, PsA or IA. The findings indicate VR may reduce risk of job loss and improve productivity, confidence managing arthritis at work, physical ability, pain and perceived health. The control group remained largely unchanged. Results should be interpreted with caution as this was a feasibility trial, with small numbers and we did not therefore conduct inferential testing.

We also conducted interviews with participants and the occupational therapists to investigate their views of VR and the work advice provided [30, 31]. Intervention group participants valued the VR, particularly the training in work-based pain and fatigue self-management, joint protection, pacing, ergonomic and job adaptations/ accommodations advice. They reported: relief from discussing work problems with the occupational therapists; making behavioural adaptations at work; and improved coping skills [30]. In contrast, the control group discussed the continuing negative impact of arthritis on their work and feeling anxious about continuing working in future. Most participants in both groups had either not read the self-help work information or considered it had little impact [30]. The occupational therapists considered: the VR was beneficial for patients; and they could now provide a more comprehensive and individualised VR service [31].

The positive quantitative and qualitative findings suggest this VR intervention is promising. Healthy work has positive health benefits [32, 33]. People with health conditions who lose their job can struggle to get back to work, have financial difficulties, reduced retirement pensions, loss of positive psychological and social support from working, reduced wellbeing and worse health. Employers lose valuable skills and health services bear additional costs [34]. Forced retirement at 65 years of age no longer exists, the UK state pension age is rising and many people will need to work for longer. Initiatives supporting helping people with arthritis stay in work for longer are of benefit to both employees and employers. Direct VR averaged under four hours and the total cost was £135/ participant (including indirect VR). If job retention VR is proven effective in an RCT, this is a relatively low cost for a potentially large saving to people with arthritis, the NHS, employers and society. In future, employer insurance could potentially pay for such services from the NHS.

The study built on previous successful job retention VR research [10], whilst making VR pragmatic and deliverable in a UK NHS setting. The intervention was individualised, flexible and spread over several months to allow participants time to make changes between sessions and reduce the impact of taking time out of work. The resource use questionnaire identified how frequently many patients have to attend other appointments, highlighting the need for flexible VR and minimising disruption to patients’ work schedules.

Whilst workplace visits are beneficial [35], we decided to make them optional as these can only be undertaken if the person has disclosed their condition to their employer and agrees to them. Accordingly, the benefits of disclosure, employer liaison and workplace visits were discussed during VR, as relevant. Only three work visits were undertaken. Most participants either did not want a workplace visit, or felt confident to address job accommodation needs with employers and a visit was not essential, which also helped reduce costs. Relatively few participants agreed to employer contact for interviews. We had anticipated higher levels of workplace engagement as national guidelines recommend a workplace component [36]. The occupational therapists delivered VR in less than the planned time and budget, with results indicating improvements in outcomes. Accordingly, providing job retention VR in these Rheumatology occupational therapy services was feasible. The control group only received written advice (reflective of that provided to many with arthritis). As no or only small effects were seen in work and health outcomes in the control group, written advice was an appropriate control intervention.

Of the candidate primary outcome measures, the absenteeism measure (monthly calendar) provided detailed data but completion rates were lower than for presenteeism and had too high a responder burden. Three monthly self-report of sick leave days (with average number of days normally worked per week) would be less onerous and could lead to better response rates. The WLQ and RA-WIS presenteeism measures are considered the best options. The Outcome Measurement in RA Clinical Trials (OMERACT) Work Group previously had identified the WLQ, WALS and RA-WIS as presenteeism measures [37, 38]. However, subsequently they determined the RA-WIS is not a presenteeism measure, although still important as a prognostic measure [39], as scores >11 are predictive of future reduction in work hours and >13 of disability leave of absence [39]. Accordingly, the WLQ is the most applicable primary outcome for an RCT. Presenteeism has the greatest impact on costs for people with RA [40], confirming the relevance of this. Sample sizes of 120 to 140 would be required for a definitive RCT with 90% power, two-sided 5% significance level and allowing for a 20% loss to follow-up, using published data on minimum clinically important differences for these presenteeism outcomes [21, 41, 42] and using the standard deviations observed in this feasibility trial.

Only 20% of people with RA consider they receive sufficient information and practical support about employment issues from their Rheumatology service [1]. Many employees with arthritis are unaware of job retention VR services available and provision of these is patchy in the UK. VR is currently available from: occupational health departments, usually only available in larger organisations, but 60% of employees work for small to medium sized enterprises; the “Fit for Work Service,” although referral to an occupational health professional is made only when an employee has been off sick for four weeks or more (before then self-help internet advice and telephone support are available) [43]; the Access to Work scheme, which the person applies for themselves, can provide grants for job accommodations (e.g. transport, equipment at work) [44]; and Job Centre Disability Employment Advisors, although services are primarily for the unemployed. NHS occupational therapists, with additional VR training, are well-placed to provide early job retention VR. Occupational analysis and therapeutic interventions in the workplace are a core part of occupational therapy practice [45]. Employed people with inflammatory arthritis experiencing work problems, can be readily identified when patients attend Rheumatology out-patients and referrals made to occupational therapy. NHS-based VR means it is also available to those who have not disclosed or do not want employer contact. This job retention VR could also be applicable for a wider range of rheumatological conditions.

During the study, some research facilitators highlighted patients, particularly in areas with higher social deprivation and in precarious employment, expressed concerns about taking time out of work and/ or did not want VR, influencing their non-participation. The British Society of Rheumatology is now supporting training for Rheumatology teams to have “work conversations” with patients to promote the importance of staying working and obtaining work advice. In a future trial, ensuring teams have training in, and implement work conversations, could prime patients to perceive VR as important to receive.

There were limitations in this study. The two groups differed in occupational groupings and, in a definitive RCT, trial stratification by occupational group (levels 1 to 4) would reduce this disparity. The recruitment time was nearly half that planned, partly due to the time obtaining R&D approvals. The NHS Health Research Authority has since streamlined approvals processes, with targets for completions and recruitment. Our recruitment strategy was screening in two Rheumatology clinics per week. However, limited research facilitator availability caused delays in sites starting, periods of no screening and early site closure. Only 10% of patients screened were randomised into the study. This was however, consistent with pre-trial predictions. A two-week audit before the trial at one site identified 25% of Rheumatology out-patients were employed people with RA, PsA or IA. Studies evaluating RA-WIS scores in employed people with RA identified 34 to 69% had scores ≥10%, i.e. moderate to high risk of work disability [11, 46–49]. We estimated nine to 18% of Rheumatology out-patients could meet key eligibility criteria, but that the other criteria and unwillingness to participate would reduce recruitment to 10% or less of out-patients.

Research facilitators had problems contacting up to 20% of those identified to complete screening or consent. Research facilitators worked only during the day and often only had patients’ home landline telephone numbers and not work/ mobile telephone numbers and e-mail addresses. This prevented contacting employed patients in the evenings. It is clearly important to ensure a range of contact details are obtained at the point of first contact with potential participants. In consequence, in a future trial, employing a research fellow, working flexibly into some evenings, would assist contacting patients outside normal work hours. The research fellow can then complete screening and consent by telephone with patients, ensure baseline questionnaires are sent out/returned and patients randomised (subject to patients’ permission for contact details to be forwarded). When research facilitators are unavailable to complete screening and recruitment, a research fellow can also complete study procedures. To increase numbers of people with RA, PsA or IA referred for screening, working age/ employed patients could be identified by healthcare team members/ research facilitators from department databases, medical and therapy notes and in therapy departments. Such patients could be mailed invitation packs by research facilitators, with interested patients returning a reply form to the research fellow who completes study procedures.

Whilst an 80% retention rate is suggested as near the limit of validity for RCTs [50], our 20% attrition was similar to other job retention VR trials [10, 51] and other RA rehabilitation trials e.g. [52, 53]. This retention rate may reflect the more adherent, motivated group of people choosing to participate and willing to commit the time, which arguably reflects the situation in every trial. The trial manager contacted participants by telephone in the evenings to give questionnaire reminders to promote retention. A research fellow could also contact participants, day and evenings, to assist questionnaire completion and obtain a minimal data set, when necessary. Additional retention strategies could be considered, such as advance postcards and text reminders indicating questionnaires will be sent shortly, a second questionnaire reminder and using financial rewards or incentives [54, 55]. We had many measures in the questionnaire, as an aim was to identify the most applicable primary work outcome measure. Accordingly, responder burden would be reduced in a definitive trial, which may encourage better completion.

## Conclusion

The findings from this study suggest job retention VR provided by NHS occupational therapists with VR training is credible, acceptable, deliverable and promising for employed people with arthritis with concerns about continuing to work in future because of their condition. The finding regarding screening and recruitment suggest recruitment could be challenging and requires additional research fellow support. Consideration needs to be given to the number needing to be screened (around 1350) to achieve the recruitment target, the number of sites and length of recruitment period required to achieve the target. There is a need for a definitive trial of this brief job retention VR in the UK, with the caveat of ensuring sufficient funding for out-of-hours and remote screening, recruitment and retention support to ensure timely recruitment and maximised response rates.

## Additional file

Additional file 1: WORK-IA Trial: contents of the: Work Self-help Information Pack; Work Rehabilitation; and Work Rehabilitation Resource Manual. **Table S6.** Self-reported health and personal resource use at six and nine months. (PDF 339 kb)

## Abbreviations

DEA: Disability Employment Advisor
EQ5D: Euroqol Five Dimensions Questionnaire
HAQ: Health Assessment Questionnaire
IA: undifferentiated inflammatory arthritis
IQR: inter-quartile range
NHS: National Health Service (UK)
NRS: numeric rating scale
OMERACT: Outcome Measures in Rheumatoid Arthritis Clinical Trials
PsA: psoriatic arthritis
QALYs: Quality Adjusted Life Years
RA: rheumatoid arthritis
RA-WIS: Rheumatoid arthritis Work Instability Scale
RCT: randomized controlled trial
SF12v2: Short Form 12 Health Survey v2
VAS: visual analogue scale
VR: vocational rehabilitation
WALS: Work Activities Limitations Scale
WES-RC: Work Experience Survey-Rheumatic Conditions
WLQ: Work Limitations Questionnaire
