# Supplementary material for: Job retention vocational rehabilitation for employed people with inflammatory arthritis (WORK-IA): a feasibility randomized controlled trial
Source: BMC Musculoskelet Disord. 2017 Jul 21;18:315. doi: 10.1186/s12891-017-1671-5 (PMC5521067; doi:10.1186/s12891-017-1671-5)
Supplement: Additional file 1: — WORK-IA Trial: contents of the: Work Self-help Information Pack; Work Rehabilitation; and Work Rehabilitation Resource Manual. Table S6. Self-reported health and personal resource use at six and nine months. (PDF 339 kb) [file 12891_2017_1671_MOESM1_ESM.pdf]

WORK-IA

Additional File 1:

WORK-IA Trial: contents of the: Work Self-help Information Pack; Vocational Rehabilitation; and Vocational Rehabilitation Resource Manual.

Work Self-help Information Pack

This was received by all participants. The control group only received this.

The participating occupational therapists s, patient research partner, working members of the Derby National Rheumatoid Arthritis Society branch (acting as study advisors) and the Trial Management Group, agreed what written information could be representative of “usual care:.

- a welcome letter, encouraging people to read the enclosed booklets and discuss work problems with relatives, friends and employers;
- a self-help flowchart, suggesting how to identify problems, resulting work difficulties and solutions to then implement and/or discuss with employers/ line managers;
- and two work self-help booklets including a summary of the Equality Act [2010], what “reasonable adjustments” employers could make, common work problems and solutions, the importance of discussing work issues with employers and colleagues and how to get further help [14,15].

Vocational rehabilitation (VR: intervention group)

VR was based on that provided in a successful VR trial in the USA [10], with content modified to suit the UK, as applicable. The occupational therapists received three days face to face VR training, (including: conducting the Work Experience Survey-Rheumatic Conditions (WES-RC) and task analysis; legislation and work services available; role play, case studies, manual handling, equipment options (e.g. seating, computer equipment), and practical workshops), plus self-study [12]. In addition, each occupational therapist was provided with a VR Resource Manual (developed for the study – see later for contents), linking problems with potential solutions. These included: solutions fact sheets, equipment recommendations, equipment sources, work station checklists and other Health and Safety Executive Guidance (e.g. on manual handling), and work-related guidance. During the study, the therapists could contact the VR trainers [RO’B, SW] for advice by telephone or e-mail.

At the first meeting, the occupational therapist used the WES-RC [16-19] to identify work problems and specific barriers (physical, psychological, environmental (physical/social) and managerial) to overcoming these. Three broad priority areas of work problems (e.g. work station modification and work positioning, moving to/around the workplace), specifying problems within these, were then collaboratively identified by the therapist and participant. The occupational therapist then recommended some initial self-management advice tailored to the participant's needs, e.g. hand exercises, relaxation, sleep hygiene, fatigue and/or joint protection advice and the occupational therapist provided appropriate booklets, e.g. Arthritis Research UK's "Keep Moving*" or "Looking After Your Joints when you have arthritis*," as applicable. The participant completed an Action Plan, with the support of the occupational therapist, with a few initial, achievable goals, to meet their identified needs, to help the participant start taking appropriate actions to resolve work problems.

Participants were asked, before the next meeting, to complete an activity diary, for 24 hours on a typical work day, identifying for each 30-minute period: their main activity; any equipment, tools or materials used; any difficulties or discomfort doing the activity; whether they took a short rest; and to rate their level of pain and fatigue using a 0 (no) to 10 (severe) scale [modified with permission; 56]. They were asked to reflect on problems identified in the WES-RC and diary and start considering possible solutions. Between the first and second meeting, the occupational therapist reviewed the three priority problem areas identified in the WES-RC and developed a range of possible solutions to discuss/ practice with the participant. At subsequent meetings, the occupational therapist and participant reviewed progress with action plans set in the previous meeting, collaboratively problem-solved, and the occupational therapist continued supporting participants to write Action Plans and in resolving difficulties for themselves. Throughout the meetings, therapists emphasised the importance of participants taking responsibility to liaise with employers to request and obtain job accommodations.

VR was individualised to include (based on the WES-RC, activity diary and priority problem areas), as applicable:

a. applying ergonomic, fatigue and stress management approaches to the workplace
b. recommendations for assistive technology/equipment adaptation, workplace/work station modification, transport advice
c. practical advice and support enabling participants to disclose their condition and negotiate job modifications with employers, e.g. discussion of strategies, how to explain their condition its impact on work, proactively make suggestions for addressing issues and role play, when applicable.
d. explaining rights under the Equality Act 2010, how and why their condition meets being one applicable under the Equality Act, what are "reasonable adjustments" employers can make in

the workplace, and the facilities available to support employers making "reasonable adjustments," e.g. Access to Work [47]; and how the participant could apply to Access to Work.

e. psychological support, through listening to and discussing work problems; encouraging ability and confidence in solving work problems, managing arthritis when working and continuing working in future
f. advice on other activities of daily living and hand function difficulties affecting work ability
g. to discuss, if necessary, about considering a change in job or career. Referral to a Disability Employment Advisor (DEA) at their local Jobcentre for advice and support in doing so, if the participant wishes
h. referral to other relevant services such as ‘mainstream' occupational therapy (e.g. for splinting, activities of daily living training, self-management education groups), physiotherapy, rheumatology or other work rehabilitation services (e.g. DEA, or advice to contact their employer’s occupational health department).
i. provision of relevant work and self-management advice booklets and other information as appropriate
j. general advice on disease flare management and contacting the Rheumatology department quickly in a flare for review and medication changes, if necessary, to reduce the risk of longer sickness absences
k. optional: if applicable, to conduct a workplace job assessment and meeting the participant and line manager together to discuss job accommodations and enabling referral to Access to Work for equipment / other support funding, as applicable.
l. A telephone review, several weeks after the final meeting, to discuss participants’ progress implementing changes, check recommended job accommodations were in place and if any further action was needed.

*Arthritis Research UK downloads available from:

Keep Moving:

http://www.arthritisresearchuk.org/shop/products/publications/patient-information/living-with-arthritis/keep-moving.aspx

Looking After Your Joints:

http://www.arthritisresearchuk.org/shop/products/publications/patient-information/living-with-arthritis/looking-after-your-joints.aspx

WORK-

WORK REHABILITATION IN INFLAMMATORY ARTHRITIS TRIAL

<u>Vocational Rehabilitation Resource Manual for OT</u>

Contents List:

1. **Research project**
    - Lecture: Work Rehabilitation in Inflammatory Arthritis: “Effectiveness and cost effectiveness of an occupational therapy job retention vocational rehabilitation intervention: a pilot randomised controlled trial”
    - Gantt chart – timeline for project
    - WORK-IA Study diagram
    - Work Rehabilitation in Inflammatory Arthritis: research protocol
    - Roles and Responsibilities of the research team/ participating therapists
    - Contact details: therapists; research team.

2. **Vocational Rehabilitation Intervention, Work Assessment Tool, VR Treatment Record, Information Pack,**
    - Summary of the Vocational Rehabilitation intervention content
    - The UK Work Experience Survey- Rheumatic Conditions (UK WES-RC): assessment [Hammond A, Woodbridge S, O’Brien R, Grant M. The UK Work Experience Survey for persons with Rheumatic Conditions (UK WES-RC). 2013. University of Salford [16].
    - UK Work Experience Survey- Rheumatic Conditions (WES-RC): Manual v2 Hammond A, Woodbridge S, O’Brien R, Grant M. The UK Work Experience Survey for persons with Rheumatic Conditions (UK WES-RC) Manual version 2. 2013. University of Salford [17].
    - Vocational Rehabilitation Treatment Record
    - The Information Pack (Intervention and Control Groups) content: Cover letter to all participants; Finding Solutions flowchart; NRAS and Arthritis Care booklets

3. Vocational Rehabilitation Training Programme

- Lectures
    a. Legislation and Policy impacting on Work
    b. Role of the Disability Employment Adviser (DEA)
    c. Rheumatoid Arthritis and Work: a service user's perspective
    d. We are not Alone! – Setting the Scene in which we work
    e. Work rehabilitation strategies
        i. Work station assessment
        ii. Work Rehabilitation strategies: the Upper Lomb
        iii. Work Rehabilitation strategies: the Environment
        iv. Load Handling
        v. Work Rehabilitation strategies: Disclosure
- Case studies: six cases (problems plus solutions)
- Example: Two completed WES-RCs, with solutions based on telephone role play activity
- Bakery case study and solutions
- Practical workshops: notes
- Peer teaching: notes/ handouts teaching how to practically do the following: conduct a worksite assessment; take micro-breaks; manage de Quervain's tendinitis; manual handling; pacing; relationships at work; disclosing arthritis at work
- Key Messages

4. Vocational Rehabilitation Solutions: Getting to Work & the Work Environment

- Travel
    a. Public transport
    b. Driving
- Access
    a. Access
    b. Flooring
        Floor protection
        Specialist flooring
    c. Emergency exit
        Personal evacuation plan
    d. Toilet

- Welfare at work – Health and Safety Executive (**HSE)** Guidance for employers on welfare provisions [http://www.hse.gov.uk/pubns/indg293.htm]

  e. Refreshments

- Working conditions
  a. Lighting
  b. Temperature
  c. Noise

5. Vocational Rehabilitation Solutions: Physical job demands

- MSD Hazards & Solutions [https://www.uwo.ca/hr/form_doc/health_safety/doc/ergo/msd_hazards_solutions.pdf]
  a) Lifting strategies:
- HSE Are you making the best use of lifting and handling aids HSE getting to grips with manual handling [http://www.hse.gov.uk/pubns/indg143.pdf]
- HSE Manual handling assessment chart (MAC) [http://www.hse.gov.uk/msd/mac/]
  a) Pushing and pulling
  b) Ladders

     HSE Safe use of ladders and step ladders [http://www.hse.gov.uk/pubns/indg455.htm]

6. Vocational Rehabilitation Solutions: Work Station Evaluation

a) Seating

   Osmond Ergonomics seating assessment chart [http://www.ergonomics.co.uk/downloads.html]

   Selecting a chair, issues for consideration

   National seating suppliers

b) Desk

   RSIA repetitive strain awareness leaflet – www.keytools.com

c) Computer monitor

   My computer My way – Abilitynet https://www.abilitynet.org.uk/

d) Keyboard

   Osmond Ergonomics range of inputting devices – keyboards (http://www.ergonomics.co.uk/)

Voice recognition software – Abilitynet: https://www.abilitynet.org.uk/

e) Mouse

Selecting a mouse

Osmond Ergonomics inputting devices, mice etc (http://www.ergonomics.co.uk/)

RSIA repetitive strain awareness – no cost computer tips (https://www.rsitips.com/; http://www.rsiprevention.com/rsi_prevention.php)

f) Wrist rest

Summary sheet

g) Document holder / writing slope

Osmond Ergonomics range of holders (http://www.ergonomics.co.uk/)

h) Laptop

Range of laptop stands

i) Writing

Range of products by EmpTech (http://www.emptech.info/)

j) Telephone

k) HSE VDU Work Station Checklist [http://www.hse.gov.uk/pubns/ck1.pdf]

7. Vocational Rehabilitation Strategies: Policies, Benefits & Legislation

- Company policies
- Benefits / assistance
    a. Disabled Living Allowance (DLA)
    b. Carers allowance
    c. Employment and Support Allowance
    d. Blue Badge scheme
    e. Work choice
    f. If an individual is in employment and becomes disabled
    g. Access to Work
    h. Legislation Summary

8. Supporting documents

- Manager support for return to work following long-term sickness absence: Guidance [Chartered Institute of Personnel Development: CIPD: https://www.cipd.co.uk/knowledge/fundamentals/relations/absence/return-to-work-guide]
- Working together? Matching work ambitions with health provision for people with arthritis – Arthritis Care
- Recruitment: an overview - CIPD [https://www.cipd.co.uk/knowledge/fundamentals/people/recruitment/factsheet]
- Disability and employment – CIPD [https://www.cipd.co.uk/knowledge/fundamentals/emp-law/disability-discrimination/factsheet]
- Harassment and bullying at work – CIPD [https://www.cipd.co.uk/knowledge/fundamentals/emp-law/harassment/factsheet]
- Managing sickness absence and return to work [Health and Safety Executive: http://www.hse.gov.uk/pUbns/priced/hsg249.pdf]
- Health Work Wellbeing : Health Care Professions' consensus statement [https://www.nhs.uk/Livewell/workplacehealth/Documents/hwwb-healthcare-professionals-consensus-statement-04-03-2008.pdf]
- College of Occupational Therapists' Vocational Rehabilitation strategy [https://www.cot.co.uk/sites/default/files/publications/public/Work_Matters_Vocational_Rehab_English.pdf]
- The Health and Work Handbook. Patient care and occupational health: a partnership guide for primary care and occupational health teams [Royal College of General Practitioners: http://www.fom.ac.uk/wp-content/uploads/hw.pdf].
- NICE Checklist to help employers and employees discuss sickness absence using NICE guidance *[*https://www.**nice**.org.uk/**guidance**/ph19/**checklist**-for-managing-**absence**-65786221]
- Fit note [https://www.gov.uk/government/collections/fit-note]
- UK Rehabilitation Council: Rehabilitation Standards [http://www.rehabcouncil.org.uk/pages/DownloadForm.aspx?Type=Standards]
- Categories for Return to Work
- HSE Ergonomics and human factors at work: http://www.hse.gov.uk/pubns/indg90.pdf

9. Relevant Articles

- Allaire S, Li W, la Valley M. Reduction of job loss in persons with rheumatic diseases receiving vocational rehabilitation. Arthritis Rheum 2003;48:3212-3218
- Allaire SH, Niu J, LaValley MP. Employment and satisfaction outcomes from a job retention intervention delivered to persons with chronic diseases. Arthritis Care Res 2005;48:3212-3218

- Tang K, Beaton DE, Gignac MAM, et al. The Work Instability Scale for rheumatoid arthritis predicts arthritis-related work transitions within 12 months. Arthritis Care Res 2010;62:1578-1587
- Allaire S, Keysor J. Development of a structured interview tool to help patients identify and solve rheumatic condition related work barriers. Arthritis Care Res 2009; 61:988-995
- Hammond A. Rehabilitation in Musculoskeletal Diseases. Best Practice Res Clin Rheum 2008;22:435-449
- Edgar J. Rheumatoid Arthritis, Work & Disability. WebMD [http://www.webmd.com/rheumatoid-arthritis/features/rheumatoid-arthritis-work-and-disability#1 Downloaded 14 October 2016].
- ES Bowers. About to Resign? Accommodations for RA at Work. http://www.webmd.com/rheumatoid-arthritis/life-with-ra-11/ra-work-accommodations
- Downloaded 14 October 2016

10. Own Notes

11. Further Resources.

12. Other information

©Alison Hammond, Rachel O'Brien, Sarah Woodbridge (2016).

Table S6: Self-reported health and personal resource use at six and nine months.

Resource	**6m: Intervention (n = 23)**	**6m: Control (n =21)**	**9m: Intervention (n = 22)**	**9m: Control (n =21)**
**Hospital service use:**				
**Contact with any hospital services (n):**	21	19	19	19
Rheumatology outpatient appointments:				
- no. attending;	21	18	16	17
- median no. visits (min-max) of attenders	2 [1-18]	3.5 [1-12]	2 [1-9]	2 [1-9]
Other appointments:				
- no. attending;	6	10	7	10
- median (min-max)	4 [1-11]	2.5 [1-30]	2 [1-6]	2 [1-3]
Day Unit attendance:				
- no. attending;	5	5	5	1
- median (min-max)	4 [2-4]	2 [1-6]	2 [1-2]	2
Accident and Emergency:				
- no. attending;	1	4	0	0
- median (min-max)	1	1.5 [1-2]		
In-patient stays:				
- no. admitted;	0	1	0	0
Rheumatology telephone helpline:				
- no. using;	6	9	6	6
- median (min-max)	2 [1-5]	2 [1-4]	1 [1-5]	2 [1-3]
Occupational Therapy appointments:				
- no. attending;	10	6	3	1
- median (min-max)	6 [1-11]	3.5 [2-4]	1 [1-2]	2
Physiotherapy appointments:				
- no. attending;	4	8	1	4
- median (min-max)	2 [1-5]	3 [1-60]	2	1 [1-2]
GP Contacts:				
- no. attending;	17	15	12	13
- median (min-max)	2.5 [1-15]	3 [1-10]	1.5 [1-4]	2 [1-4]
District and other Nurse appointments:				
- no. attending;	9	7	4	5
- median (min-max)	6 [1-26]	2 [1-6]	3 [1-6]	1 [1-2]
**Personal service use:**				
Home-help/cleaner (paid)				
- no. using;	2	0	2	1
- median contacts (min-max)	64 [24-104]	0	32 [12-52]	12 [12-52]

**Unpaid help from friends/relatives:**				
Help with childcare:				
- no. using;	2	0	1	0
- median hours/week (min-max)	2	0	3	0
Help with personal care:				
- no. using;	6	8	3	9
- median hours/week (min-max)	2 [2-9]	2.5 [1-8]	3 [2-4]	5.5 [1-14]
Help in and around the house:				
- no. using;	10	13	9	11
- median hours/week (min-max)	9.5 [2-26]	5 [3-20]	12 [1-35]	15 [4-30]
Help outside the house:				
- no. using;	9	8	7	8
- median hours/week (min-max)	3 [1-9]	3 [2-6]	5 [4-20]	8 [2-30]
